# Pulmonary hypertension as seen in a rural area in sub-Saharan Africa: high prevalence, late clinical presentation and a high short-term mortality rate during follow up

**DOI:** 10.5830/CVJA-2018-007

**Published:** 2018

**Authors:** Dzudie Anastase, Suiru Dzekem Bonaventure, Martin, Dzudie Anastase, Suiru Dzekem Bonaventure, Ndemnge Aminde Leopold, Abanda Martin, Dzudie Anastase, Dzudie Anastase, Sliwa Karen, Tantchou Tchoumi Cabral, O Mocumbi Ana, Pascal Kengne Andre, Thienemann Friedrich, Sliwa Karen, Ndemnge Aminde Leopold

**Affiliations:** Departments of Internal Medicine and Physiology, Faculty of Medicine, University of Yaoundé, Yaoundé, Cameroon; Departments of Internal Medicine and Physiology, Faculty of Medicine, University of Yaoundé, Yaoundé, Cameroon; Departments of Internal Medicine and Physiology, Faculty of Medicine, University of Yaoundé, Yaoundé, Cameroon; Douala General Hospital and Clinical Research Education, Networking and Consultancy, Douala, Cameroon; Douala General Hospital and Clinical Research Education, Networking and Consultancy, Douala, Cameroon; Douala General Hospital and Clinical Research Education, Networking and Consultancy, Douala, Cameroon; Douala General Hospital and Clinical Research Education, Networking and Consultancy, Douala, Cameroon; Soweto Cardiovascular Research Group, Department of Medicine, University of the Witwatersrand, Johannesburg, South Africa; NIH Millennium Fogarty Chronic Disease Leadership Programme; NIH Millennium Fogarty Chronic Disease Leadership Programme; Shisong Cardiac Centre, Kumbo, Cameroon; Instituto Nacional de Saúde, and Faculty of Medicine, Eduardo Mondlane University, Maputo, Mozambique; Non-communicable Diseases Unit, South African Medical Research Council, Cape Town, South Africa; Clinical Infectious Diseases Research Initiative, Institute of Infectious Diseases and Molecular Medicine, Faculty of Health Science, University of Cape Town, Cape Town, South Africa; Hatter Institute for Cardiovascular Research in Africa, Faculty of Health Sciences, University of Cape Town, Cape Town, South Africa; School of Public Health, Faculty of Medicine and Biomedical Sciences, University of Queensland, Brisbane, Australia

**Keywords:** pulmonary hypertension, prevalence, mortality, Shisong, Cameroon

## Abstract

**Introduction:**

The epidemiology of pulmonary hypertension (PH) in low- to middle-income countries is poorly characterised. We assessed the prevalence, baseline characteristics and mortality rate in patients with echocardiographically diagnosed PH at a rural cardiac centre in Cameroon.

**Methods:**

We conducted a prospective cohort study in a subsample of 150 participants, aged 18 years and older, diagnosed with PH [defined as right ventricular systolic pressure (RVSP) ≥ 35 mmHg in the absence of pulmonary stenosis and right heart failure]. PH was classified as mild (RVSP: 35–50 mmHg), moderate (RVSP: 51–60 mmHg) and severe (RVSP: > 60 mmHg).

**Results:**

Of 2 194 patients screened via echocardiograms, 343 (crude prevalence 15.6%) had PH. The sub-sample of 150 patients followed up (54.7% women, mean age of 62.7 ± 18.7 years) had a mean RVSP of 68.6 mmHg. They included 7.3% mild, 29.3% moderate and 63.4% severe PH cases. Co-morbidities included log smoke (80.7%), hypertension (52.0%), family history of cardiovascular disease (50.0%), diabetes (31.3%), alcohol abuse (21.3%) and HIV infection (8.7%). Main clinical features were dyspnoea (78.7%), fatigue (76.7%), palpitations (57.3%), cough (56.7%), jugular venous distension (68%) and peripheral oedema (66.7%). Overall, 70% presented in World Health Organisation functional class III/IV. PH due to left heart disease (PHLHD) was the commonest (64.7%), and rheumatic valvular disease accounted for 36.1%. The six-month mortality rate was 28%.

**Conclusion:**

PH, dominated by PHLHD, was common among adults attending this rural centre and was associated with a high mortality rate. Related co-morbidities and late clinical presentation reflect the poor socio-economic context. Improved awareness of PH among physicians could promote early diagnosis and management.

Pulmonary hypertension (PH) is defined as an increase in mean pulmonary arterial pressure (mPAP) at or above 25 mmHg at rest.^1^,^2^ It is currently classified by the World Health Organisation (WHO) into five subtypes, which include pulmonary arterial hypertension (PAH), PH due to left heart disease (PHLHD), PH due to lung disease or hypoxia (PHLDH), chronic thromboembolic PH (CTEPH) and PH due to unclear or multifactorial mechanisms (PHUM).^3^,^4^

The prevalence of pulmonary vascular disease in the developing world is unknown, but estimates suggest that about 25 million individuals may be affected.^5^ Little information exists on the epidemiology of PH in sub-Saharan Africa, however there is some evidence that based on the high prevalence of risk factors such as rheumatic heart disease, schistosomiasis and HIV infection in this area of the world, the prevalence and mortality rate of PH may be higher than in Western countries.^6^,^7^

In South Africa, PH has been identified as one of the commonest causes of death, accounting for 31% of total cardiovascular deaths,^8^ while only 8% of cardiovascular deaths in the United Kingdom were attributed to PH in 2012.^9^ Furthermore, studies from the United States have shown that the prevalence of PH among African Americans is higher than in Caucasians.^10^ These differences in the epidemiology of PH in different regions of the world are determined by genetic, geographic, environmental and socio-economic factors.

Left heart disease has been widely suggested to be the most common cause of PH. In developing countries, chronic infectious diseases, hypertensive heart diseases, cardiomyopathy and rheumatic heart disease are the main contributors.^6^ This study aimed at determining the prevalence, baseline clinical characteristics and mortality rate during six months of follow up of patients with PH diagnosed via echocardiography at the rural Shisong Cardiac Centre (SCC) in Cameroon.

## Methods

This was a prospective cohort study in a sub-sample of 150 participants aged 18 years and older who were diagnosed with PH via echocardiography. It was conducted at the Shisong Cardiac Centre from September 2013 to December 2014. This study also forms part of the Pan-African Pulmonary Hypertension Cohort study (PAPUCO).

Shisong is a rural village in the Kumbo sub-division of the north-west region of Cameroon. Shisong, on the outskirts of Kumbo town, is about 400 km north of Douala, the economic capital of Cameroon, and 450 km north-west of Yaounde, the political capital of Cameroon.^11^ The Shisong Cardiac Centre (SCC) is a well-equipped centre for the diagnosis and management of a variety of cardio-surgical conditions including PH. On average 185 echocardiographic examinations are done per month. In this study, the target population was restricted to patients living in rural or sub-urban areas, aged 18 years and above, who underwent echocardiographic examination at the centre between September 2013 and December 2014.

The PAPUCO study design and procedures have been described in detail elsewhere.^12^ In brief, PH was diagnosed using echocardiography in patients with a right ventricular systolic pressure (RVSP) ≥ 35 mmHg in the absence of acute right heart failure (HF) and pulmonary stenosis. The datacollection form, adapted from the PAPUCO study, was used to obtain patients’ information and clinical characteristics, including socio-demographic factors and past medical history [age, gender, body mass index (BMI), HIV status, family history of cardiovascular disease, systemic hypertension, dyslipidaemia, smoking and alcohol consumption], clinical presentation (dyspnoea, cough, fatigue, pedal oedema, palpitations and World Health Organisation functional classification).

At six months post-baseline, patients and/or their next-of-kin were contacted by phone to determine their vital status. For all fatal outcomes, the probable cause of death was assessed through a verbal autopsy.

## Statistical analysis

Data were analysed using SPSSR (Statistical Package for Social Sciences for Windows) version 20. Qualitative variables are summarised as frequencies and percentages. Continuous variables are represented as means and standard deviations, or median (25th to 75th percentiles). Patients were categorised in three groups depending on PH severity; mild if RVSP was 36–50 mmHg, moderate if RVSP was 51–60 mmHg and severe if RVSP was > 60 mmHg. We used χ2 to compare proportions and Student’s t-test or Kruskal–Wallis test to compare mean differences for continuous variables. Statistical significance was accepted at a p-value of 0.05.

## Results

**Fig. 1:**
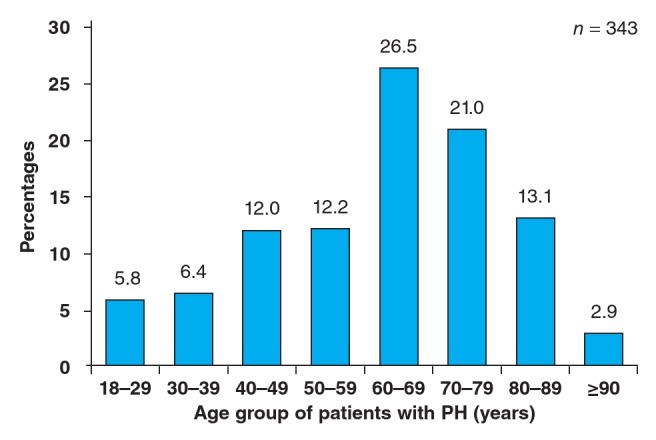
Age distribution of patients with pulmonary hypertension in the Shisong Cardiac Centre.

Out of a total of 2 194 patients who underwent cardiac echography at baseline, 343 had PH (prevalence rate 15.6%). Mean age was 61.9 ± 18.0 years and female gender (189, 55.1%) was predominant. As shown in Fig. 1, the peak prevalence of PH was noticed between 60 and 69 years (91/343, 26.5%).

Characteristics of the sub-sample followed up (n = 150) were similar to those of the overall PH group. The mean baseline age was 62.7 years [standard deviation (SD) =18.7]. Mean age did not vary significantly by severity of PH (p = 0.25). Most participants (44.7%) had primary education, 32% had secondary education while 15.3% had never been to school. Variations by severity of PH were not significant (p = 0.69). The 150 followed-up participants included 11 (7.3%) with mild PH, 44 (29.3%) with moderate PH and 95 (63.4%) with severe PH. The proportion of women was 54.7% overall, and 5, 20 and 57%, respectively among the mild, moderate and severe PH groups (p = 0.09).

**Fig. 2:**
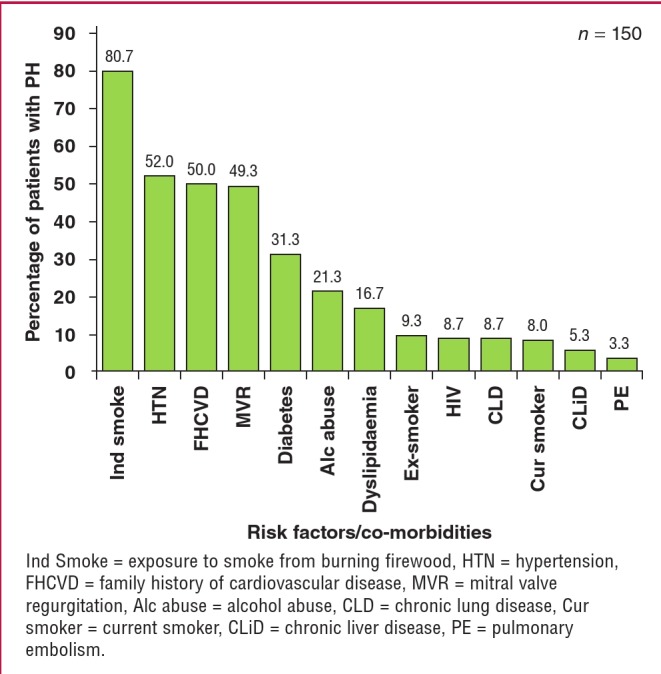
Risk factors and co-morbidities among 150 patients with pulmonary hypertension followed up at the Shisong Cardiac Centre.

The distribution of risk factors for PH and co-morbidities are depicted in Fig. 2. Exposure to cooking fumes (80.7%), systemic hypertension (52.0%), family history of cardiovascular disease (50.0%), mitral valve regurgitation (49.3%), diabetes (31.3%) and alcohol abuse (21.3%) were the most common factors and co-morbidities identified in our study participants.

Dyspnoea (78.7%), fatigue (76.7%), palpitation (57.3%) and non-productive cough (56.7%) were the main symptoms reported by patients on initial presentation. Syncope (6.7%) and cyanosis (6.0%) were rarely reported by our patients. Most patients who participated in this study had distended jugular veins (68.0%) and peripheral oedema (66.7%). Table 1 summarises variations in clinical signs and symptoms with PH severity. Chest pain varied significantly with PH severity (p = 0.03)

**Table 1:** Clinical and echocardiographic findings of adult patients with PH

*Parameters*	*All (n = 150)*	*Mild PH (n = 11)*	*Moderate PH (n = 44)*	*Severe PH (n = 95)*	*p-value*
Clinical features at presentation					
Difficulty breathing (dyspnoea), n (%)	118 (78.7)	9 (7.6)	32 (27.1)	77 (65.3)	0.32
Cyanosis, n (%)	9 (6.0)	1 (11.1)	2 (22.2)	6 (66.7)	0.58
Non-productive cough, n (%)	85 (56.7)	10 (11.8)	22 (25.9)	53 (62.3)	0.14
Fatigue, n (%)	115 (76.7)	8 (7.0)	30 (26.0)	77 (67.0)	0.10
Syncope, n (%)	10 (6.7)	0	3 (30.0)	7 (70.0)	0.33
Palpitations, n (%)	86 (57.3)	8 (9.3)	28 (32.6)	50 (58.1)	0.06
Chest pain, n (%)	49 (32.7)	6 (12.2)	17 (34.7)	26 (53.1)	0.03
Distended jugular veins, n (%)	102 (68.0)	8 (7.8)	26 (25.5)	68 (66.7)	0.22
Peripheral oedema, n (%)	100 (66.7)	9 (9.0)	25 (25.0)	66 (66.0)	0.43
NYHA I and II, n (%)		45 (30.0) 3 (6.0)	19 (42.2)	23 (51.1)	0.13
NYHA III and IV, n (%)	105 (70.0)	8 (7.6)	25 (23.8)	72 (68.6)	0.13
Vital signs					
BMI (kg/m^2^)	26.3 (18–46.8)	23.9 (21.2–26)	26.2 (18.6–42.4)	27.1 (18–46.8)	0.03
Systolic BP (mmHg)	126 (65–250)	133 (102–190)	123 (95–235)	127 (65–250)	0.26
Diastolic BP (mmHg)	79 (45–154)	73 (58–106)	78 (60–154)	80 (45–130)	0.73
Heart rate (beats/min)	88 (52–150)	96 (80–119)	88 (52–120)	86 (56–150)	0.43
Respiratory rate (breaths/min)	23 (13–40)	22 (19–28)	22 (13–35)	23 (15–40)	0.20
O2 saturation (%)	93 (55–100)	90 (82–98)	94.5 (67–99)	91.5 (55–100)	0.37
Echographic parameters					
LVEDD (mm)	53 (16–72)	36 (18–56)	50 (38–70)	55 (16–72)	0.0001
LVESD (mm)	42 (13–60)	35 (13–43)	42 (22–60)	42 (18–97)	0.003
Ejection fraction (%)	48 (20–91)	66 (32–91)	46 (32–72)	46 (20–88)	0.06
Fractional shortening (%)	23 (6–95)	49 (28–61)	29 (18–33)	21 (6–95)	0.09
TAPSE (mm)	10 (7–25)	11 (8–20)	10 (8–17)	10 (7–25)	0.70

Data are number (%) or median (IQR).

BMI = body mass index, O2 = oxygen, LVEDD = left ventricular end-diastolic diameter, LVESD = left ventricular end-systolic diameter, TAPSE = tricuspid annular plane systolic excursion.

**Fig. 3:**
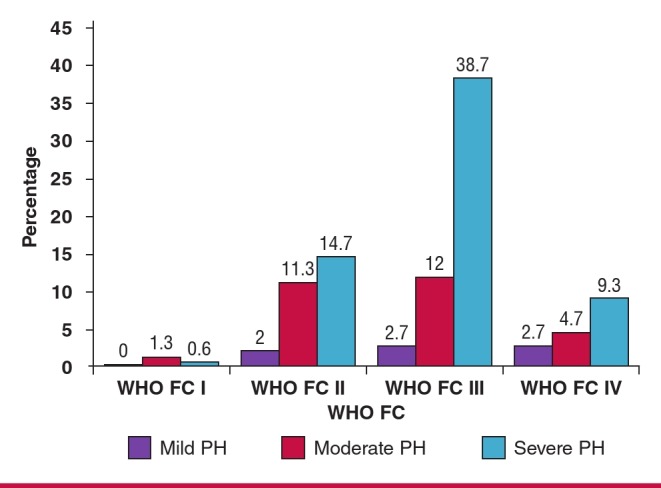
Distribution of patients across WHO functional classes and PH severity.

Fig. 3 shows variations of the World Health Organisation functional class (WHO FC) according to PH severity. More than half (53%) of the patients presented in WHO FC III, 28% presented in class II, while 17 and 2% presented in class IV and I, respectively. Therefore a greater proportion of patients presented with marked functional limitation.

**Fig. 4:**
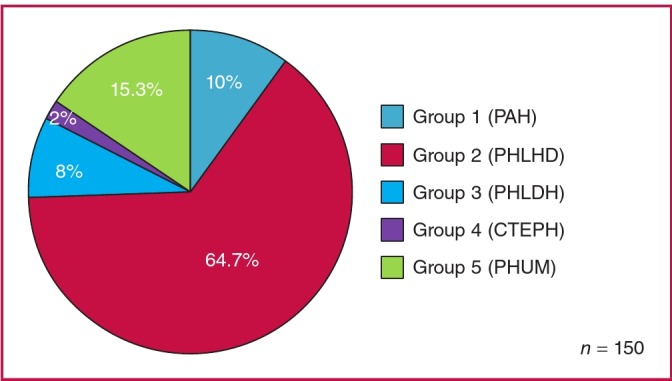
Patient distribution according to the updated clinical classification of PH.

The main cause of PH was left heart disease (group 2), accounting for 64.7% of all cases, as shown in Fig. 4. In addition, 15.3% was due to unclear or multifactorial aetiology (group 5), 10% due to pulmonary arterial hypertension (group 1), 8% due to lung disease or hypoxia (group 3), and only 2% due to chronic thromboembolic mechanisms (group 4). Out of 97 participants with PHLHD, 50.5% had left ventricular systolic dysfunction (heart failure with reduced ejection fraction, HFrEF: EF ≤ 50%), 36.1% had valvular heart disease and 13.4% had left ventricular diastolic dysfunction (HFpEF: EF > 50%).

The duration of follow up of the 150 participants ranged from five to 180 days. After a median follow up of 90.5 days, 42 deaths (cumulative mortality rate of 28%) were recorded. Equivalent figures were five deaths (cumulative incidence 45.5%) in mild PH, nine deaths (cumulative incidence 20.5%) in moderate PH and 28 deaths (cumulative incidence 29.5%) in severe PH (p = 0.28).

## Discussion

Our study aimed at determining the prevalence, clinical profile and mortality rate from PH in a rural setting in sub-Saharan Africa. We noted a high prevalence of PH, late presentation to healthcare facilities in an advanced state of heart failure, and consequently a high mortality rate at six months of follow up. These findings could be attributed to the poor socio-economic status, hyper-endemicity of risk factors for PH, and limited availability of PH-specific drug therapies. In the PAPUCO study,^7^ which was a multinational study on the epidemiology of PH in Africa with recruitment centres mostly in urban areas, similar findings were noted. Therefore it can be said that PH still presents a challenge on the African continent overall and not only in the rural setting.

Our observed prevalence of 15.6% is higher than the average of 10% prevalence observed in Australia in 2012 and in other European countries.^13^ This is somewhat to be expected considering the high burden of risk factors such as rheumatic heart disease, schistosomiasis, tuberculosis, sickle cell disease and HIV infection in sub-Saharan Africa, in addition to other risk factors shared with high-income countries. In addition, the SCC is located in a rural area that is difficult to access. Therefore, patients are usually reluctant to visit the centre until they are in advanced disease states or when referred by cardiologists. A recent expert review on the global perspective of the epidemiology of PH also supports our findings.^6^ Among the several co-morbidities assessed in our study population, exposure to cooking fumes was the most common, especially in women. This most likely results from the common practice in Africa and Cameroon, particularly in the rural setting, where women cook using open fires, unlike in high-income countries. Systemic arterial hypertension was also common and in line with studies from Africa,^7^ USA^14^,^15^ and Germany.^16^

Hypertension is very common in sub-Saharan Africa where it affects about 30% of the adult population, and mostly goes undetected, undertreated and inadequately controlled.^17^ It is the principal cause of HF in sub-Saharan Africa. In the Pan-African THESUS-HF registry of HF for instance, it was estimated that up to 50% of HF cases were due to uncontrolled hypertension.^18^ This high prevalence of uncontrolled hypertension would most likely also account for the high proportion of PHLHD in our study population. With the growing epidemic of HF, LHD is now globally recognised as the main cause of PH.^6^,^7^,^13^ PHLHD was dominated by patients with left ventricular systolic dysfunction, while PH due to rheumatic valvular heart disease is still common in our setting.

The clinical presentation was dominated by exertion dyspnoea, fatigue, cough and palpitations, which are common and non-specific symptoms in most patients with cardiovascular and/or respiratory conditions. Study participants were slightly overweight with a mean BMI higher than observed in a study in Nigeria,^19^ but lower than reported in the USA.^15^ Most of our participants presented with moderate to severe functional limitation, with 70% of them presenting in WHO FC or New York Heart Association (NYHA) class III and IV.

These findings are similar to those in the PAPUCO study,^7^ and to those of Baptista and colleagues in Portugal in 2013,^20^ who observed that 71% of their patients presented in WHO FC III and IV, as well as those of Fikret and colleagues in Germany.^16^ This global observation of late presentation to medical attention could be explained by the fact that most symptoms and signs of PH are non-specific and therefore cases are usually misdiagnosed in primary care until the later stages when patients seek specialist care. Furthermore, in Africa, poor access to healthcare, limited availability of diagnostic tools for PH, and the general reluctance of patients in rural settings to seek medical attention until the later stages of illness could explain at least in part the late presentation.

About a third of our patients died within the first six months of being diagnosed with PH. This mortality rate is three times higher than that observed in the USA15 and the UK.^9^ The high mortality rate in our setting is most likely accounted for to some extent by the unavailability of disease-specific drug therapies. The fact that patients present at an advanced stage of the disease, and their inability to comply with follow-up visits reflects to some extent their limited financial coping capacity, resulting in death in the absence of adequate care.

## Limitations

Our study has some limitations. Some cases of PH could have been missed because indications for cardiac echocardiography are usually symptom driven. This would lead to over-diagnosis of patients with severe disease, and accordingly, poor outcomes. Therefore whether our finding reflects those of a typical population with PH in this setting is unknown. Diagnosis of PH in our study was done by echocardiography, which is more a screening tool for PH, while right heart catheterisation (RHC), which is the gold standard for diagnosing PH, was not used. Therefore, cases of mild PH could have been missed in our study. Furthermore the operator-dependent nature of echocardiography could lead to over- or under-diagnosis. Despite the fact that echocardiography is only a screening tool, it is paramount in the diagnosis of PH as it is non-invasive, more available and less expensive compared to RHC. Moreover, in expert hands, it yields reliable and reproducible results. Indeed, studies carried out to evaluate the diagnostic accuracy of echocardiography compared to RHC have demonstrated a sensitivity of 83% and a specificity of 72%.^21^

## Conclusion

Our findings suggest that PH is very common among patients attending our rural cardiac centre, with PHLHD being the most frequent type, and the short- to medium-term mortality rate being excessively high. Patients tend to present in advanced stages of disease and usually with several co-morbidities, most of which are cardiovascular conditions. Healthcare practitioners in this setting should be made more aware of this devastating condition, in order to prompt timely referral to specialised centres for proper evaluation and care of patients with suspected PH.
